# Variability in Profiles and Prevalences of Gram-Negative Bacteria in Urinary Tract Infections: A Population-Based Analysis

**DOI:** 10.3390/jcm13175311

**Published:** 2024-09-07

**Authors:** Carlotta Nedbal, Nitin Mahobia, Dave Browning, Bhaskar Kumar Somani

**Affiliations:** 1 Department of Urology, University Hospital Southampton NHS Trust, Southampton SO16 6YD, UK; 2 Urology Unit, ASST Fatebenefratelli-Sacco, 20157 Milan, Italy; 3 Department of Infection, University Hospital Southampton NHS Trust, Southampton SO16 6YD, UK; nitin.mahobia@uhs.nhs.uk (N.M.);

**Keywords:** gram negative, urinary tract infections, antimicrobial resistance, antibiotic stewardship

## Abstract

**Objective:** An increase in antimicrobial resistance (AMR) is observed worldwide, partly due to the overuse and misuse of antibiotics, which are ineffective in certain population subgroups. This negatively impacts both the healthcare system and patients. Our study aimed to investigate the current AMR profiles for the most commonly used antibiotics in treating urinary tract infections (UTIs) caused by gram-negative bacteria (GNB) across different age and gender subpopulations. By doing so, we provide valuable information for doctors managing prophylactic and empiric therapeutic treatments. **Materials and Methods:** We retrospectively analysed over 650,000 urine cultures collected in the Microbiology Department of a referral university hospital in Southern England from January 2014 to December 2022. A population-based analysis for subgroups was performed to rule out differences in AMR patterns. Our report was recorded at UHS as an internal audit (UHS7670). **Results:** 146,867 cultures were found positive for GNB growth. Nitrofurantoin showed the best sensitivity patterns for all age subgroups (0.93% for patients aged ≤ 18; 1.22% for patients aged 19–40; 2.17% for patients aged 40–60; and 3.48% for patients aged > 60), regardless of gender (male: 6.37%, female: 2.59%). Ampicillin/amoxicillin and trimethoprim showed a poor AMR profile for all age groups (>55% and >28%, respectively) and genders (>60% and >28%, respectively). All the other tested antibiotics (cefalexin, cefotaxime, ceftazidime, ciprofloxacin, co-amoxiclav, gentamicin) showed an overall good profile for GNB resistance across all subgroups. For all antibiotics except trimethoprim, the risk of developing AMR was significantly higher in the male population. We also found that people aged over 60 had a higher risk of AMR compared to the other age groups for all antibiotics, with the exception of cefotaxime and co-amoxiclav. **Conclusions:** With an overall rise in resistance patterns for GNB-related UTIs, certain antibiotics—particularly ampicillin/amoxicillin and trimethoprim—now exhibit very poor sensitivity profiles. However, antibiotics such as nitrofurantoin and gentamicin remain excellent options for empirically treating UTIs. It is important to note that AMR can vary across different populations, with higher resistance often found in elderly and male patients. Clinicians must stay informed about current guidelines and research to provide the best treatment options while minimizing the risk of further AMR development.

## 1. Introduction

Antimicrobial resistance (AMR) poses a growing global health challenge, creating challenges in managing intricate urinary tract infections (UTIs) due to the reduced efficacy of commonly prescribed antibiotics [1]. The increase in antibiotic (AB) resistance showed by prevalent pathogens is linked to the global overuse of ABs, a fact which leads to escalating difficulties in the empirical treatment of complicated UTIs. UTIs related to gram-negative bacteria (GNB), a family known for its ability to escape AB treatment and develop new resistances [2], have a significant impact on AMR and healthcare. This carries medical consequences in hospital facilities, including possible severe effects on intensive care unit (ICU) recoveries and contributing to heightened mortality and morbidity [3].

The practice of antibiotic stewardship has therefore been included in and promoted by national and international guidelines on AB use, emphasizing the important role of narrow-spectrum ABs in reducing the selection of microbial strains and, consequently, mitigating the rise in multi-resistant infections, often associated with the use and abuse of wide-spectrum ABs [4]. AB stewardship programmes involve strategically selecting, evaluating, and controlling antimicrobial treatments—with the aim of properly optimizing clinical outcomes and eradicating or preventing infectious diseases—while achieving optimal clinical outcomes in infection treatment or prevention and diminishing the possible patient-related harm due to AMR development. The role played by AB stewardship programmes is pivotal in promoting the prudent use of ABs, thus reducing avoidable overuse and optimizing the cost-effectiveness of therapies [5].

To maintain effective treatment and appropriate AB selection, it is crucial to continuously monitor susceptibility trends in local, high-volume centers. The danger of selecting an ineffective AB and thus promoting pathogen resistance is a significant concern, with potentially harmful consequences for patients [6]. AMR complicates the treatment of complex UTIs, often requiring extended and more intensive treatment, which can adversely affect both patients and healthcare systems [7]. Furthermore, the growing prevalence of multi-resistant microbial strains found in urine specimens heightens the risk that commonly used antibiotics may fail to act swiftly in preventing sepsis, especially in high-risk populations [8].

In light of the growing emphasis on AB stewardship programs and the rising impact of multi-resistant microbial species on our healthcare system, the importance of regular reassessment of data on AB resistance and sensitivity is paramount. To address this need, we conducted a retrospective analysis of patterns in gram-negative bacteria antimicrobial resistance against the most frequently used ABs in a high-volume university hospital over the last decade. Our study aims to provide an updated AMR profile across different age groups and genders, offering insights into the most effective treatment options tailored to diverse patient characteristics.

## 2. Materials and Methods

From January 2014 to December 2022, all urine samples for culture analysed in the microbiology department at University Hospital Southampton (UHS) were collected and retrospectively analysed for the present study. Our research was conducted in the Microbiology Department of the UHS Foundation Trust, UK. With a reservoir of almost 4 million people living in the region, UHS experiences a high turnover of patients, making it one of the major National Health Service (NHS) trusts.

Throughout the cited timeline, culture specimens were continuously collected as part of routine procedures, with each sample’s origin (primary or secondary care), type (catheter bag, mid-stream urine), patient characteristics (age, gender), and collection date being recorded. If a patient provided more than one urine culture within a three-month period (e.g., for recurrent UTIs), the second specimen was excluded from our analysis to prevent redundant data collection.

Each sample was tested for pathogen growth at the time of arrival at the microbiology laboratory. For the purpose of the research, the following GNB were selected for inclusion: *Acinetobacter*, *Escherichia coli*, *Klebsiella*, *Morganella*, *Serratia marcescens*, *Pseudomonas* and *Proteus* (in alphabetic order) [9]. Eleven antibiotics were chosen for antimicrobial resistance and sensitivity analysis, in line with the guidelines from the National Institute for Health and Care Excellence (NICE) and the European Association of Urology (EAU). These antibiotics, listed in alphabetical order, are as follows: ampicillin/amoxicillin, cefalexin, cefotaxime, ceftazidime, ciprofloxacin, co-amoxiclav, gentamicin, nitrofurantoin, and trimethoprim.

We conducted an analysis of urine samples to detect pathogen growth, primarily utilizing the semi-automated MAST urine culture method. The MAST URI^®^SYSTEM (www.mast-group.com/uk/products/mast-uri-system, accessed on 15 August 2024) is a semi-automated platform designed for the direct culture, identification, and antibiotic susceptibility testing (AST) of urinary pathogens. Through direct media inoculation, image analysis, and advanced software, the system enables the reporting of approximately 95% of results within 24 h. To further assess resistant organisms, we employed the disc sensitivity test according to the European Committee on Antimicrobial Susceptibility Testing (EUCAST) standards, utilizing 96-well plates for sensitivity from the MAST URI^®^SYSTEM. Our microbiology laboratory is accredited by the United Kingdom Accreditation Service (UKAS), the foremost accreditation authority in the UK, and consistently maintained full UKAS accreditation throughout the study period. At the microbiology laboratory of the UHS we follow the EUCAST guidelines, as is common practice in most European laboratories. The EUCAST antibiotic breakpoint tables, updated annually since Version 1.0 in 2010 through to Version 13.0 in 2023, were used for susceptibility testing, with the most current version applicable to the year of AB sensitivity testing being utilized. It was deemed appropriate to keep the EUCAST breakpoint of each current year for the respective samples, rather than reviewing the analysis secondarily at the time of the study.

Over the nine-year period, all analysed samples were compiled into an Excel database (Excel 2021, Microsoft Office Professional Plus 2021, Microsoft Corporation, Redmond, WA, USA) and categorised by positivity and pathogen characteristics. AB sensitivity data for each GNB-UTI was recorded and subsequently analysed using XLSTAT (XLSTAT 2023 statistical software for Microsoft Excel, Lumivero, Denver, CO, USA). We performed a population-based analysis, first labelling samples according to the patient’s gender (male, female) and secondarily according to age (<18 years old, between 19 and 40, between 41 and 60, and over 60 years old). For each group, we performed a correlation analysis using odds ratios (ORs) and *p* values, applying a threshold of 0.005. Significant ratios of antimicrobial resistance for the specific population and antibiotics where proposed and discussed with an interdisciplinary team involving urologists, microbiologists, and infectious disease experts to review current protocols for AB management. Our results were compared to the guidelines to check for any needed revision.

As the present study included only retrospective and descriptive analysis of strictly anonymised data, assigned random serial codes to the specimens, and did not affect the clinical evaluation and treatment of patients included, we did not need an ethical board approval. Nevertheless, the study was registered as an audit (US7670) at UHS, and the relevant data are available from the registry.

## 3. Results

Over 650,000 urine samples were collected in the Microbiology Department during the 9 years of the analysis period. Of them, 146,867 cultures were found to be positive for GNB growth. 46,029 (31.34%) urine samples were sent from a secondary care facility (specialist care, consultant/referral care, hospitals), 98,023 (66.74%) from primary health care facilities (general practice, family medicine, community healthcare). Samples sent from different facilities not under primary or secondary care were not categorised. Most of the collected samples were obtained routinely from midstream urine (133,620, 90.98%), while catheter samples accounted for a small part (13,247, 9.02%). Most of the patients providing samples were female (119,830, 81.59%), with only 18% being male patients (27,037). The majority of patients were elderly, with 57.21% aged over 60. Patients aged less than 18 years represented only 8.75% of the population. Table 1 summarises these population characteristics.

The spectrum of AB microbial sensitivity and resistance was analysed according to the standard EUCAST breakpoint-based methods for each sample. Percentages of resistance to specific antibiotics were reported for each subgroup.

Gender analysis revealed significant differences in resistance spectra between the genders (Table 2), with increased risk of resistance in the male population for all the antibiotics with the exception of trimethoprim (OR: 0.928, *p* = 0.232). Trimethoprim showed a relevant resistance pattern in both genders, with an overall percentage of resistance over 28%. GNB also showed high resistance towards ampicillin/amoxicillin, with 60.30% resistance in the female population and 71.64% resistance in the male population. In the male population, two other antibiotics were found with a borderline-high level of resistance: cefalexin (19.43%) and ciprofloxacin (15.03%). All the other tested antibiotics showed a good profile for both groups, with still better sensitivity spectra found in the female population. Our analysis confirmed a very low resistance percentage for nitrofurantoin, followed by cefotaxime, ceftazidime, co-amoxiclav and gentamicin.

An analysis of resistance patterns among different age subgroups was performed with the elderly group as a reference, the results of which are displayed in Table 3. Overall, AMR was confirmed with results similar to those found in the gender analysis, with ampicillin/amoxicillin and trimethoprim showing high levels of resistance in all age subgroups: over 55% in all age groups for ampicillin/amoxicillin and over 28% for trimethoprim.

In the youngest group (aged ≤18), the best AMR was found for nitrofurantoin (0.93%, OR: 0.33). Cefalexin (9.60%, OR: 0.87), ceftazidime (4.07%, OR: 0.72), ciprofloxacin (6.36%, OR: 0.49), and gentamicin (4.64%, OR: 0.77) also showed an excellent AMR, with reduced risk of resistance compared to the elderly population. Cefotaxime (3.90%, OR: 1.18) and co-amoxiclav (4.33%, OR: 1.19) also had very good AMR profiles but a slightly higher risk of resistance compared to the reference population.

For people aged between 19 and 40 and between 41 and 60, very similar results were found. Nitrofurantoin showed the best AMR (1.22%, OR: 0.39; and 2.17%, OR: 0.68, respectively). The risk of AMR was lower for both populations compared to the oldest group for cefalexin (9.46%, OR: 0.71; and 9.54%, OR: 0.79, respectively), ceftazidime (3.88%, OR: 0.65; and 4.27%, OR: 0.73, respectively), ciprofloxacin (7.03%, OR: 0.58; and 8.33%, OR: 0.75, respectively), and gentamicin (4.33%, OR: 0.92; and 4.39%, OR: 0.84, respectively).

## 4. Discussion

UTIs are a common and significant health concern in the UK, placing a substantial burden on the healthcare system. Despite this, no studies have been conducted in Hampshire, a region with a population exceeding one million. This study seeks to examine the epidemiology of UTIs and the trends in antimicrobial resistance in Southampton, a city in southern England. Although several studies have been conducted to investigate the prevalence and patterns of AMR in different regions, there is still a lack of scientific evidence on the matter of population-specific patterns. With the present study, we provided a first evaluation of the different trends in GNB-UTI antibiotic susceptibility according to age and gender.

Our report found that most UTI cases (82%) occurred in females, consistent with previous research [10]. The increased vulnerability of women to UTIs can be ascribed to anatomical factors, such as a shorter urethra, along with hormonal and behavioural influences [11]. Several factors have been associated with the development of GNB-UTIs worldwide and in the UK, and need to be considered during treatment to evaluate possible multi-resistant germs. Here, these infections have been shown to be linked to indwelling devices, such as ureteral stents, nephrostomies, urinary catheters or vascular accesses. Moreover, GNB-UTIs can be correlated with recent exposure to invasive and minimally invasive procedures—particularly prostate biopsies, endoscopies, and gastrointestinal surgeries—but are also related to recent hospitalisations or AB therapies, as well as haematological disorders such as neutropenia [12].

GNB constitute a significant portion of the bacteria accountable for AMR, possibly because of their well-known capacity to evade therapeutical strategies [13]. Innately, GNB harbour resistance mechanisms, such as efflux pumps and porin mutations, which augment their ability to endure antimicrobial agents [14]. As possible proof of the presence of an escaping mechanism in GNB, our study found a good sensitivity profile for co-amoxiclav, while the resistance to amoxicillin was overall high at >50% in all age groups. This might reflect the production of beta-lactamases by the bacteria and their ability to mutate to overcome antibiotics efficacy. The heightened prevalence of multidrug-resistant (MDR) organisms among GNB species, such as *Escherichia coli*, *Klebsiella pneumoniae*, and *Pseudomonas aeruginosa*, underscores the urgent need for effective screening methods to protect the efficacy of current treatment options [15].

Our results indicated that GNB from UTIs are mostly susceptible to nitrofurantoin, gentamicin, co-amoxiclav, ceftazidime, and cefotaxime. Decades ago, nitrofurantoin initially emerged as a highly effective and safe antibiotic prescribed for UTIs. However, over time, several bacterial pathogens have developed resistance to it [16]. The prevalence of nitrofurantoin resistance has shown variability over the years, ranging from 10% to over 70% [17]. Despite its long-term use, numerous studies have documented low bacterial resistance (0–5%) in most parts of the world [18]. These results are in line with ours, reporting a very low resistance profile showed by GNB towards nitrofurantoin in all age populations. Nitrofurantoin appears to be particularly beneficial for UTI treatment due to its high concentration in urine [19], making it a favourable choice for managing uncomplicated cystitis, which is characterized by a generally low resistance level. Given its effectiveness and minimal associated risks, nitrofurantoin has understandably gained widespread use, although it is important to note that this wide usage could potentially lead to a more extensive resistance trend [20].

As described in the EAU guidelines, ciprofloxacin is a first-line treatment for pyelonephritis [21]. Our findings support its use for UTIs, though resistance tends to increase with age and is higher in males. Reduced use of quinolones for uncomplicated UTIs might explain the low overall AMR [22]. Gentamicin, a secondary treatment for pyelonephritis and the first choice for urosepsis [23], showed robust sensitivity in both males and females, with low resistance even among the elderly. EAU guidelines suggest co-amoxiclav for combination therapy or based on urine culture sensitivity patterns [24], while NICE guidelines recommend it for parenteral treatment of pyelonephritis in non-pregnant individuals over 16 years old [25]. Our analysis found co-amoxiclav effective across age groups, with slightly higher resistance in males but a low overall AMR.

A recent study conducted by Kazmi and colleagues [26], which analysed AMR in 137 patients with UTIs in Saudi Arabia, found that nitrofurantoin, fosfomycin, and amoxicillin-clavulanate are the best first-line oral empiric antimicrobial drugs for adult patients presenting with UTIs in this region. Similarly, trimethoprim and ciprofloxacin were not recommended. Negri and colleagues [27] investigated GNB-related UTIs in Brazil, where *P. mirabilis* was found to be responsible for most paediatric infections, while *P. aeruginosa* and *E. faecalis* were the most frequent pathogens in the over 65 age group. Again, they discussed nitrofurantoin and fosfomycin as the most appropriate treatment for uncomplicated UTI, reserving amoxicillin as a second line and aminoglycosides or carbapenems for treating pyelonephritis. These findings align with ours, showing a possible changing pattern in different geographical areas that is possible due to antibiotic overuse. A different picture was drawn by Fu and colleagues [28], who investigated different GNB infections in 18 emergency departments in China. UTI isolated cultures provided information on treatment susceptibility, revealing amikacin, tobramycin, and meropenem to be the most effective antibiotics.

A promising new approach for UTI treatment involves the use of immuno-prophylactic vaccines and various nanotechnology solutions, such as nanoparticles (NPs) [29]. NPs can serve as delivery systems for drugs targeting specific sites. Additionally, nanotechnology offers the potential to develop enhanced nano-antibiotics by incorporating different NPs, like gold and copper, into their structure. The potential of NPs was investigated by Mekky and colleagues [30] in a recent study that indicates that biosynthesized silver NPs exhibit promising antimicrobial and antioxidant properties against UTI pathogens, including strains resistant to multiple antibiotics. However, NP drugs are not currently tested and certified, and further research is warranted to ensure effectiveness and safety for in vivo use [31].

The escalating trend of AMR poses a global healthcare challenge, significantly impacting both primary and secondary care. The widespread and at times inappropriate use of antibiotics—constituting up to 20–50% of all prescribed antibiotics in acute care [32]—has contributed to a growing resistance pattern against commonly employed antibiotics. Thus, the crucial role of National and International guidelines in prescribing appropriate first-line treatments cannot be overemphasized. Countries such as the UK, USA, South Africa, Colombia, and Australia have implemented antimicrobial stewardship programs in their healthcare systems [33], involving infection specialists and various health professionals—including nurses, community health workers, and pharmacists—to address the global population’s needs.

The use of antibiotics in animals is another concerning issue, reported as a possible origin for human MDR infections. Colistin, a new last-line antibiotic for UTIs, was reported to encounter fast resistance from *E. coli* species, due to the presence of plasmid-coded, colistin-resistance genes derived from animals and then transferred to humans via horizontal transmission [34]. Momani and colleagues investigated the prevalence of colistine-resistant GNB-related UTIs in Jordan [35], finding a concerningly rising number of resistant pathogens. They addressed the crucial need to robustly utilize antibiotics to control and prevent the emergence and prevalence of colistin-resistance genes.

Our research centres on the population of Hampshire, where we analysed a large number of urine cultures collected at our university hospital. This high-volume study, encompassing hundreds of thousands of GNB-positive samples, offers valuable insights into current clinical practices. It is imperative that antibiotic stewardship be fully integrated into both primary and secondary care [36]. The first empirical treatment decisions, often made by general practitioners or physicians in care homes and community settings, are crucial for selecting the most appropriate ABs for suspected, clinically-significant UTIs [37]. Overprescribing antibiotics for asymptomatic bacteriuria can be harmful, particularly for patients without risk factors and those with recurrent UTIs, as it may contribute to higher rates of AMR [38,39]. When treating clinically significant infections, it is vital to consider the common resistance patterns of frequently encountered bacteria, especially in populations with risk factors such as indwelling catheters, ureteric stents, recent surgeries, or prior antibiotic use.

While acknowledging its limitations, this study offers significant insights into the evolving trends in AMR through the analysis of urine cultures from a varied patient cohort. The retrospective nature of the research and its focus on a single tertiary hospital may limit the applicability of the findings to a broader population. Additionally, some important details, such as risk factors that might influence the growth of resistant GNB, were not consistently recorded for all patients, leading to their exclusion from our analysis. The retrospective nature of the present study indeed limited certain aspects of the investigation, such as the lack of a timely analysis of patient characteristics, including the pivotal difference between community-acquired and hospital-acquired UTIs and the correlated impact on the clinical management of these patients. Similarly, we were not able to reconstruct the origin and past medical history of the patients—so as to categorize them into complicated, uncomplicated, and recurrent UTIs—or to perform a thorough analysis of separate uro-pathogen resistance that takes into account the patients’ characteristics. Further investigation is required to explore other contributors to UTI development, including host immune responses, bacterial virulence factors, and patient demographics. Moreover, there is a recognized potential for bias, as many patients with uncomplicated UTIs could have been treated effectively with empiric therapy in community settings, bypassing the need for urine cultures to identify pathogens and assess antibiotic sensitivity. This suggests that the actual prevalence of GNB-related UTIs in the broader population might vary. Nonetheless, considering the study’s large cohort size and the comprehensive resistance data included, we believe our findings provide a reliable reflection of the current AMR landscape in southern England.

## 5. Conclusions

With an overall increase in resistance patterns for GNB-related UTIs, some antibiotics now show a very poor sensitivity profile (ampicillin/amoxicillin, trimethoprim). Other antibiotics still represent an excellent option for empirically treating UTIs, in particular nitrofurantoin, gentamicin, ciprofloxacin, and co-amoxiclav. Keeping in mind that AMR might differ among different populations, with higher resistance to be found in the elderly and male populations, clinicians must stay up-to-date with current guidelines and research to provide the best treatment options while reducing the risk of further AMR.

## Figures and Tables

**Table 1:** Population demographics. The demographic characteristics of the collected specimens were recorded. Gender differences are shown in the rows, while age groups are separated into columns.

	Age Group				
Gender	≤18	19–40	41–60	>60	Total
Male	1623 (6.00%)	1455 (5.38%)	4457 (16.48%)	19,502 (72.13%)	27,037 (18.41%)
Female	11,231 (9.37%)	23,180 (19.34%)	20,904 (17.44%)	64,515 (53.84%)	119,830 (81.59%)
Total	12,854 (8.75%)	24,635 (16.77%)	25,361 (17.27%)	84,017 (57.21%)	146,867

**Table 2:** Analysis of AMR patterns according to population gender.

Antibiotic	Category	Resistance n (%)	OR (CI)	*p* Value
Amp/Amoxicillin	Male	19,371 (71.64%)	1.663 (1.616–1.712)	<0.001
	Female	72,261 (60.30%)	Reference	
Cefalexin	Male	5252 (19.43%)	1.957 (1.889–2.027)	<0.001
	Female	13,143 (10.97%)	Reference	
Cefotaxime	Male	1989 (7.48%)	1.592 (1.510–1.678)	<0.001
	Female	5694 (4.82%)	Reference	
Ceftazidime	Male	1931 (7.31%)	1.130 (1.006–1.199)	<0.001
	Female	5438 (4.63%)	Reference	
Ciprofloxacin	Male	4024 (15.03%)	1.644 (1.581–1.708)	<0.001
	Female	11,523 (9.71%)	Reference	
Co-amoxiclav	Male	2186 (8.23%)	1.734 (1.647–1.825)	<0.001
	Female	5786 (4.91%)	Reference	
Gentamicin	Male	2059 (7.70%)	1.571 (1.492–1.655)	<0.001
	Female	5973 (5.03%)	Reference	
Nitrofurantoin	Male	1705 (6.37%)	2.551 (2.401–2.771)	<0.001
	Female	3080 (2.59%)	Reference	
Trimethoprim	Male	7603 (28.41%)	0.928 (0.901–0.956)	0.232
	Female	35,531 (29.93%)	Reference	

OR: odds ratio; CI: confidence interval.

**Table 3:** Comparative analysis of AMR in four different age groups for each antibiotic tested.

Antibiotic	Age Group	Resistance n (%)	OR (CI)	*p* Value
Amp/Amoxicillin	≤18	6295 (56.05%)	0.788 (0.757–0.821)	<0.001
	19–40	13,056 (56.32%)	0.793 (0.769–0.818)	<0.001
	40–60	12,312 (58.90%)	0.886 (0.859–0.914)	<0.001
	>60	40,598 (62.93%)		
Cefalexin	≤18	1078 (9.60%)	0.873 (0.807–0.946)	<0.001
	19–40	2004 (9.46%)	0.708 (0.664–0.755)	<0.001
	40–60	1994 (9.54%)	0.789 (0.743–0.837)	<0.001
	>60	8067 (12.50%)		
Cefotaxime	≤18	438 (3.90%)	1.182 (1.028–1.360)	<0.001
	19–40	881 (3.95%)	1.405 (1.257–1.571)	<0.001
	40–60	873 (4.18%)	1.229 (1.110–1.361)	<0.001
	>60	3502 (5.43%)		
Ceftazidime	≤18	457 (4.07%)	0.722 (0.659–0.792)	<0.001
	19–40	866 (3.88%)	0.651 (0.606–0.700)	<0.001
	40–60	892 (4.27%)	0.778 (0.728–0.832)	<0.001
	>60	3664 (5.68%)		
Ciprofloxacin	≤18	716 (6.36%)	0.491 (0.452–0.533)	<0.001
	19–40	1568 (7.03%)	0.584 (0.550–0.620)	<0.001
	40–60	1741 (8.33%)	0.745 (0.705–0.786)	<0.001
	>60	7498 (11.62%)		
Co-amoxiclav	<18	486 (4.33%)	1.194 (1.051–1.356)	0.001
	19–40	886 (3.97%)	1.018 (0.918–1.129)	<0.001
	40–60	916 (4.38%)	1.053 (0.959–1.157)	<0.001
	>60	3498 (5.42%)		
Gentamicin	≤18	409 (3.64%)	0.773 (0.699–0.855)	<0.001
	19–40	961 (4.33%)	0.917 (0.852–0.987)	<0.001
	40–60	917 (4.39%)	0.843 (0.786–0.905)	<0.001
	>60	3686 (5.71%)		
Nitrofurantoin	≤18	105 (0.93%)	0.330 (0.282–0.387)	<0.001
	19–40	279 (1.22%)	0.393 (0.351–0.440)	<0.001
	40–60	454 (2.17%)	0.676 (0.620–0.737)	<0.001
	>60	2242 (3.48%)		
Trimethoprim	≤18	3218 (28.65%)	1.139 (1.089–1.192)	<0.001
	19–40	6030 (35.16%)	0.965 (0.932–1.000)	<0.001
	40–60	5998 (28.69%)	0.990 (0.957–1.025)	<0.001
	>60	20,285 (31.44%)		

OR: odds ratio; CI: confidence interval.

## Data Availability

Data are available upon request from the authors.
